# Bullet Wound Blues: A Case Report on Brachial Artery Pseudoaneurysm

**DOI:** 10.7759/cureus.42408

**Published:** 2023-07-24

**Authors:** Anjali S Mannava, Ana c Braga, Ayda Fathollahpour, Niharika Ryali, Milenko Lazarevic

**Affiliations:** 1 Department of Medicine, All India Institute of Medical Sciences, Raipur, IND; 2 Department of Medicine, University of Brasilia, Brasilia, BRA; 3 Department of Medicine, Iran University of Medical Sciences, Tehran, IRN; 4 Department of Medicine, Gandhi Medical College, Hyderabad, IND; 5 Department of Internal Medicine, Swedish Covenant Hospital, Chicago, USA

**Keywords:** proximal humerus, humerus, gunshot wound, brachial artery, pseudoaneurysm

## Abstract

Pseudoaneurysms are typically iatrogenic due to the increasing use of the artery for arterial interventions such as invasive vascular radiological procedures, invasive coronary artery procedures, arterial punctures for an arteriogram, or catheterization. Other reasons for pseudoaneurysm formation are intravenous drug use and penetrating trauma. They are more commonly observed in the lower limbs than in the upper limbs. In this case report, we present the occurrence of a brachial artery pseudoaneurysm (BAP) in a 73-year-old male patient who suffered a gunshot wound (GSW) 25 years ago during the war and was admitted to the hospital because of a fall episode. This case represents one of the few documented instances of a pseudoaneurysm formation following a GSW in the United States. Along with that, we describe the subsequent medical care provided to the patient.

## Introduction

Pseudoaneurysms, also known as false aneurysms or communicating hematomas, present as pulsatile masses. Brachial artery pseudoaneurysms (BAPs) are infrequent and can occur as a result of gunshot wounds (GSWs). In such instances, the injury often leads to a rupture in the arterial wall, accumulating blood outside the vessel in an adjacent cavity and eventually forming a hematoma. The management of these pseudoaneurysms is not standardized and depends on factors such as size and symptoms, with treatment options ranging from surgical to conservative approaches. However, it is crucial to carefully evaluate the patient and administer appropriate treatment. Otherwise, developing a false aneurysm can have detrimental consequences [1].

## Case presentation

A 73-year-old male presented to the emergency departed following a fall. He had a history of coronary artery disease (CAD), non-ST elevation myocardial infarction (NSTEMI), cardiomyopathy, hyperlipidemia, hypertension (HTN), systolic congestive heart failure (CHF), and atrial fibrillation (AF) with a pacemaker. An electrocardiogram (ECG) revealed sinus rhythm with premature atrial complexes, aberrant conduction, left axis deviation, and left ventricular hypertrophy with QRS widening and repolarization. In physical examination, he had normal vital signs with a blood pressure of 135/93 mmHg and normal neurological examination with no signs of stroke. The investigation revealed a mass in the patient's left arm, at the same site where he had a gunshot wound over 25 years ago during the war. At that time, the bullet passed through the arm ultimately. He has been taking the following medications: apixaban, clopidogrel, Entresto, metoprolol succinate, pantoprazole, simvastatin, spironolactone, and a vitamin D supplement. Regarding the past surgical history, there were cardioversion and coronary angioplasty, both in 2019.

**Figure 1:**
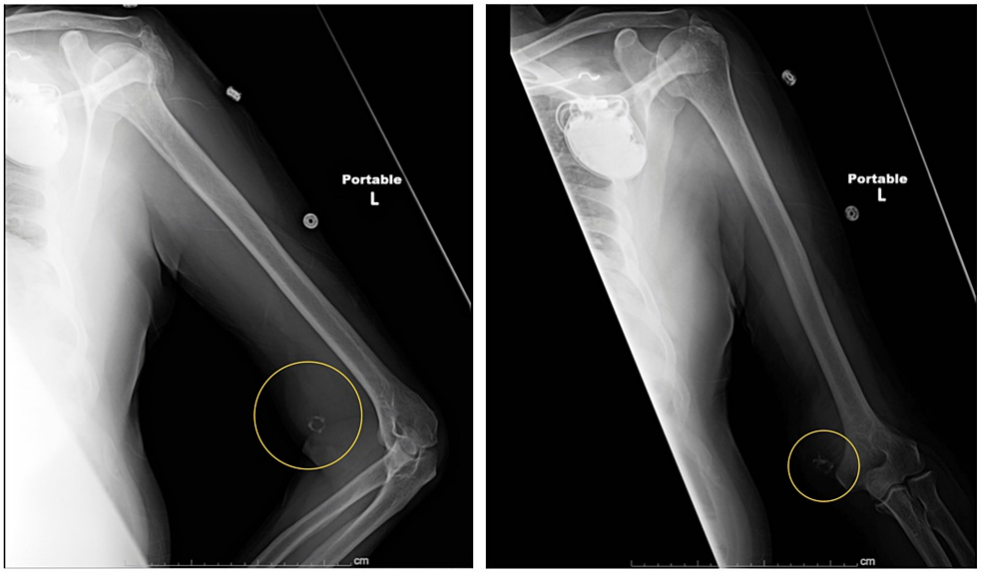
X-ray in AP view showing calcified density within possible mass in the distal upper arm (marked in yellow). AP: anteroposterior

**Figure 2:**
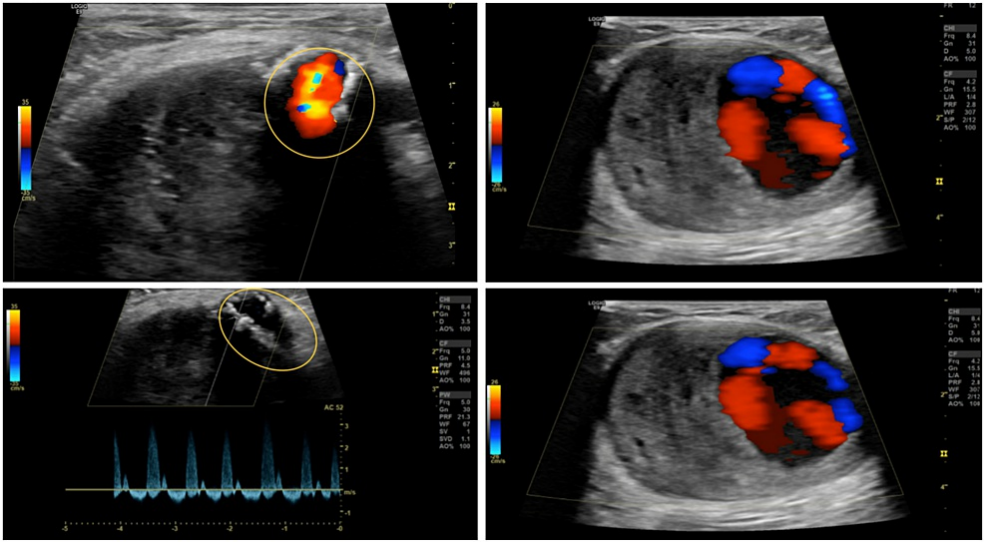
Ultrasound of the left extremity confirmed a 6 cm partially thrombosed pseudoaneurysm arising from the left brachial artery (false lumen marked in yellow).

**Figure 3:**
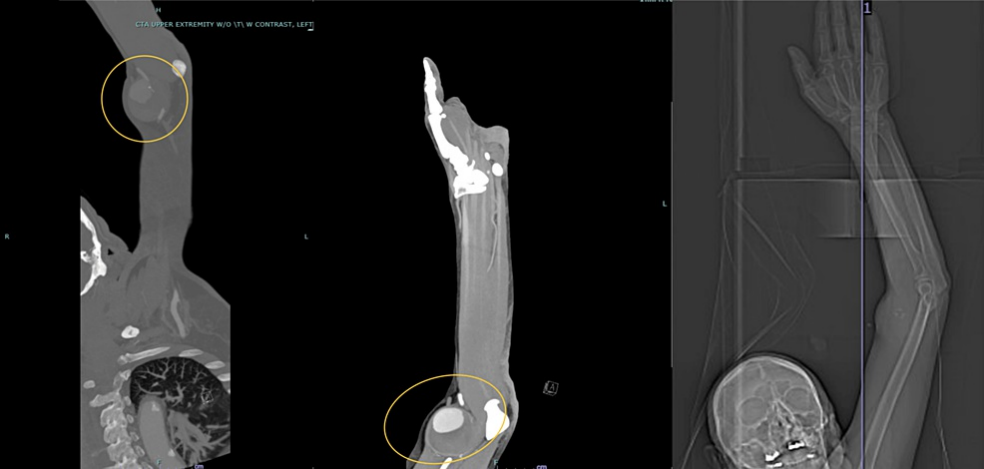
CTA of the left upper extremity revealed a 5.2 × 6.4 × 4.9 cm partially thrombosed pseudoaneurysm in the distal aspect of the brachial artery, just proximal to the elbow joint. There is a calcified neck at the origin of the pseudoaneurysm, measuring about 1 cm in diameter (marked in yellow). CTA: computed tomography angiogram

X-ray of the humerus identified a calcified density within a possible mass in the distal upper arm without affecting the underlying humeral bone (Figure 1). Ultrasound showed a false lumen in the distal brachial artery (Figure 2). A computed tomography angiogram (CTA) of the left upper extremity revealed a 5.2 × 6.4 × 4.9 cm partially thrombosed pseudoaneurysm in the distal aspect of the brachial artery, just proximal to the elbow joint. There is a calcified neck at the origin of the pseudoaneurysm, measuring about 1 cm in diameter (Figure 3).

Due to severe pain and the risk of impending rupture, the patient underwent repair of the left brachial artery pseudoaneurysm. The normal brachial artery was identified proximally and distally during the procedure, and each was encircled with a vessel loop. The pseudoaneurysm was opened to determine the arterial lumen, allowing for complete dissection. The contents were sent for histopathology analysis, and the brachial artery was reapproximated without tension using a 6-0 prolene suture.

Outcome and follow-up

The postoperative phase was uneventful. The patient was discharged two days after the surgery. Four weeks later, the patient was presenting with normal function and sensation in the left arm.

## Discussion

Pseudoaneurysms of the upper limb vessels are indeed much less common than those in the lower limbs [2]. They can occur in arteries throughout the body due to various factors, including infection, rupture of an existing aneurysm, congenital diseases (such as Ehlers-Danlos syndrome, mycotic aneurysms, Kawasaki disease, osteochondromas, polyarteritis nodosa, Menkes disease, and Behçet disease, among others), surgical procedures, or penetrating trauma [3]. Pseudoaneurysms resulting from penetrating trauma have been reported to occur in less than 0.5% of patients, and brachial artery pseudoaneurysm (BAP) is a particularly rare condition, with an occurrence rate of less than 0.04% of cases [4,5]. Weakening of the vessel walls following penetrating trauma is a possible underlying mechanism for the development of pseudoaneurysm later in life. Therefore, understanding the factors contributing to its development and identifying appropriate treatment strategies are crucial.

Several risk factors have been associated with the development of pseudoaneurysms. These include the use of anticoagulants or antiplatelet drugs, obesity, diabetes, large sheath size, improper puncture technique, inadequate manual compression, arterial hypertension, difficulties in compressing puncture sites, severely calcified arteries, and hemodialysis [6,7]. Treatment options for pseudoaneurysms vary depending on the size of the pseudoaneurysm (those that are larger than 2 cm require surgical repair), the associated symptoms, its location, and pathogenesis. Surgery may be needed in rare cases; most of them are managed using injections, ultrasound-guided compression, observation, or minimally invasive procedures. Conservative management with observation may be appropriate for small (less than 2 cm), asymptomatic pseudoaneurysms, while larger or symptomatic pseudoaneurysms often require intervention. Treatment modalities can include surgical repair, endovascular procedures such as coil embolization or stenting, ultrasound-guided compression, or thrombin injection [1]. Ultrasound-guided compression can be used in brachial arteries. It is a cost-effective and safe technique [8].

A study by Delf et al. investigated the factors associated with pseudoaneurysm development and the need for future reintervention. They found that a pre-procedural platelet count below 150 × 10^9^/L, pseudoaneurysms that arose after a vascular surgical procedure, and the use of arterial closure devices (ACDs) during the causative pseudoaneurysm procedure were more likely to require future interventions [9].

However, we have many articles describing treatment options for femoral artery pseudoaneurysms. The literature is still not wide and precise about the specific treatment of brachial pseudoaneurysms.

## Conclusions

Pseudoaneurysm formation in the brachial artery following a gunshot wound is a rare but significant complication that requires prompt identification and appropriate management. While conservative approaches such as compression or thrombin injection may be sufficient for small, asymptomatic pseudoaneurysms, larger or symptomatic pseudoaneurysms pose a higher risk of rupture and often necessitate surgical or endovascular intervention. Understanding the risk factors associated with pseudoaneurysm development is crucial for early detection and appropriate treatment selection.

In this case report, our patient presented with both pain and a high risk of rupture of the brachial artery pseudoaneurysm. Therefore, surgical treatment was chosen to address the condition and ensure favorable patient outcomes. Further research and exploration of alternative therapeutic approaches are warranted to enhance our understanding and optimize management strategies for this rare complication.
